# Gas sensing properties of nanocrystalline diamond at room temperature

**DOI:** 10.3762/bjnano.5.243

**Published:** 2014-12-04

**Authors:** Marina Davydova, Pavel Kulha, Alexandr Laposa, Karel Hruska, Pavel Demo, Alexander Kromka

**Affiliations:** 1 Institute of Physics, Academy of Science of the Czech Republic, Cukrovarnicka 10, 16200 Prague, Czech Republic; 2 Department of Microelectronics, Faculty of Electrical Engineering, CTU in Prague, Technicka 2, 16627 Prague, Czech Republic

**Keywords:** gas sensor, integrator, interdigitated electrodes, nanocrystalline diamond, response

## Abstract

This study describes an integrated NH_3_ sensor based on a hydrogenated nanocrystalline diamond (NCD)-sensitive layer coated on an interdigitated electrode structure. The gas sensing properties of the sensor structure were examined using a reducing gas (NH_3_) at room temperature and were found to be dependent on the electrode arrangement. A pronounced response of the sensor, which was comprised of dense electrode arrays (of 50 µm separation distance), was observed. The sensor functionality was explained by the surface transfer doping effect. Moreover, the three-dimensional model of the current density distribution of the hydrogenated NCD describes the transient flow of electrons between interdigitated electrodes and the hydrogenated NCD surface, that is, the formation of a closed current loop.

## Introduction

Air pollution is one of the main environmental-health related threats. Increasing amounts of noxious pollutants are emitted into the atmosphere, resulting in damage to human health and the environment. There is great interest in using sensing devices to improve the environmental and safety regulations of toxic gases in particular within buildings, underground structures, and airports.

Semiconducting solid-state gas sensors can be considered as the most promising, portable, miniaturized gas sensors because of their minimal power requirements. However, most of these sensors show poor selectivity, and the main drawbacks are slow sensor response times and recovery speeds. Even if the response time for the detection of reactive gases is rapid (e.g., within the range of seconds), in many cases, the recovery times at room temperature can be from several hours up to several days. Only thermal annealing of the sensor reduces the sensor recovery speed [1–2].

Recently, much attention has been given to solid-state integrating-type (i.e., accumulating- or dosimeter-type) gas sensing devices, which are able to overcome the aforementioned drawbacks [3–7]. To date, various publications have focused on conductometric integrating gas sensors, which are able to avoid several problems of conventional gas sensors. Nevertheless, the proper choice of the sensing material plays an essential role [3–4].

Diamond is a promising sensor material and can be deposited on the existing sensor elements. Undoped diamond is an extremely good insulator and upon the formation of a covalent bond with hydrogen, exhibits semiconducting properties [8]. This conductivity arises from free positive charge carriers (holes) in the sub-surface region and is highly sensitive to surrounding gases and/or liquids. Our previous works have shown that interdigitated electrodes (IDEs) capped with nanostructured, hydrogen-terminated, nanocrystalline diamond (NCD) were very sensitive and selective, especially towards phosgene gas [9–10]. In addition, we observed that the sensor sensitivity was strongly dependent on the total surface area.

In this study, we demonstrate a gas sensor based on hydrogen (H)-terminated NCD with an integrating measurement principle. The influence of the surface area (adjusted by nucleation time) and the electrode arrangement on the sensitivity of the NCD sensor is discussed. Finally, a simulation of the distribution of the current density of H-terminated NCD is presented.

## Results and Discussion

### Hot plasma microwave PECVD system

Microwave plasma chemical vapor deposition is a well-established process for the fast growth of high quality diamond. In the present study, the hot plasma system with an ellipsoidal, cavity-like reactor was used to grow diamond films under the following conditions: sample temperature, 450 °C; power, 1 kW; gas pressure, 30 mbar; hydrogen and methane flow rates, 300 and 3 sccm (i.e., 1% dilution), respectively; and deposition time, 5 h.

**Figure 1:**
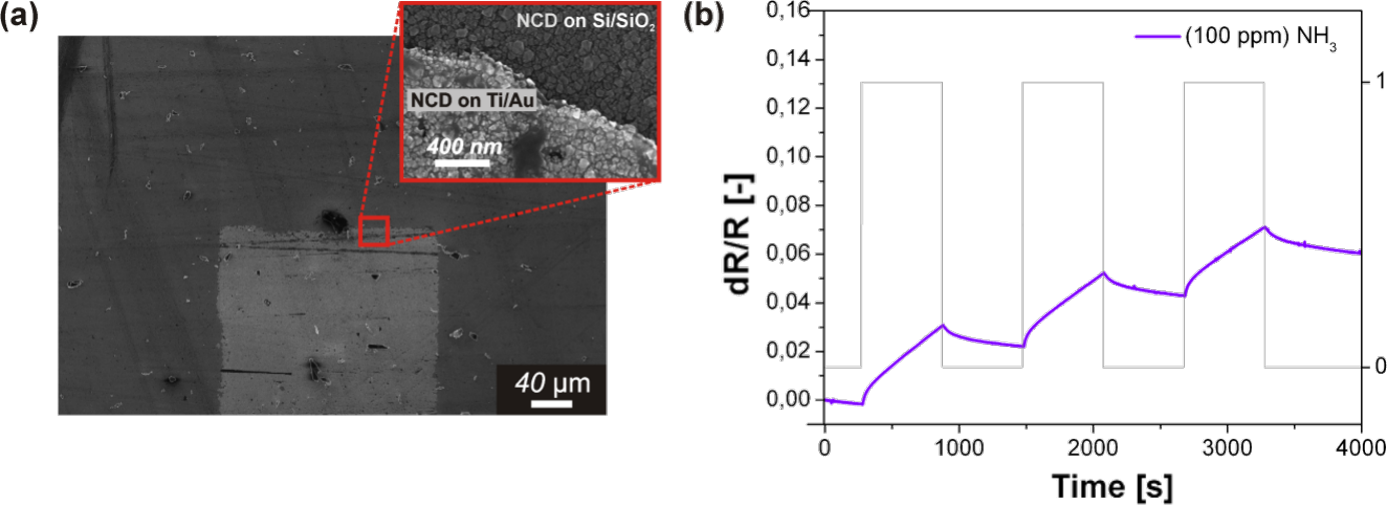
(a) SEM surface morphology of an NCD-coated sensor substrate with IDEs with separation of 200 µm and a nucleation time of 40 min, and (b) corresponding plot of the sensor response vs time.

Figure 1a shows the SEM image of the surface morphology of the sensor substrate (Si/SiO_2_ + IDEs with a separation of 200 µm) coated with the NCD layer using a 40 min nucleation time. This top view depicts the presence of an amorphous carbon shell at the diamond grains (film) and a visible development of diamond nanocrystal faceting. Moreover, the NCD primarily grew on the IDEs as was previously found [9].

The gas-sensing properties of the hydrogenated NCD sensor with a sparse electrode arrangement of 200 µm were tested against a sequence of NH_3_ pulses (Figure 1b).

These results demonstrated that the hydrogenated diamond sensor exhibited a clear response to each sequence of NH_3_, and this behavior indicated that the H-terminated NCD sensor demonstrated an integrator-type gas response.

**Figure 2:**
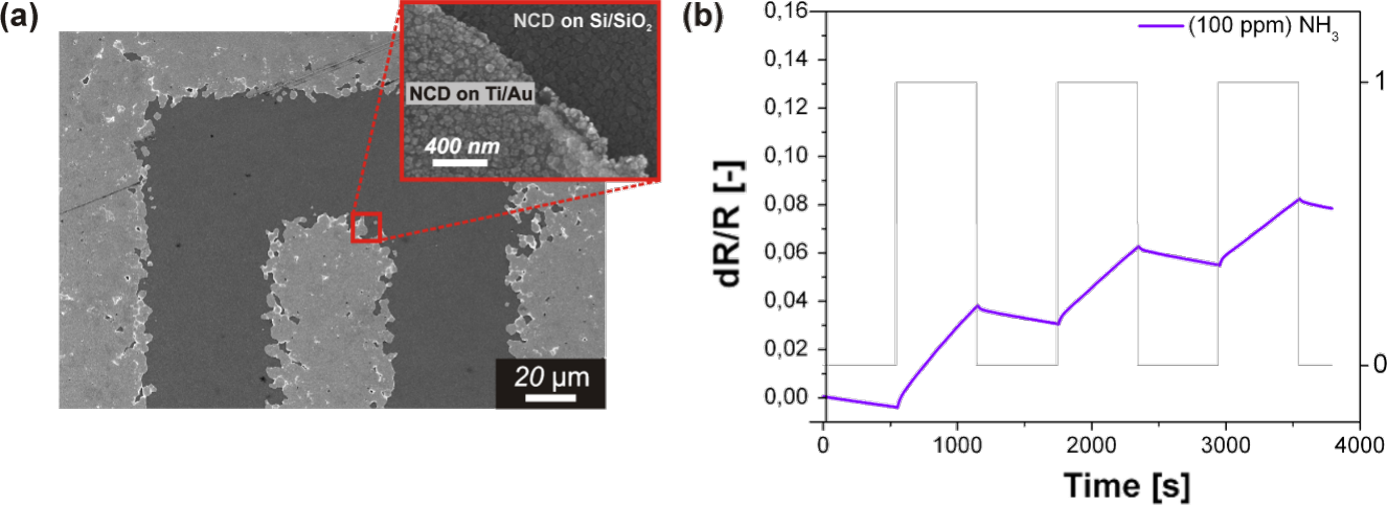
(a) SEM surface morphology of an NCD-coated sensor substrate with IDEs with separation of 50 µm and a nucleation time of 40 min, and (b) corresponding plot of the sensor response vs time.

Figure 2a shows an SEM image of the surface morphology of the sensor substrate (Si/SiO_2_ + IDEs with a separation of 50 µm) coated with the NCD layer after 40 min of nucleation time. The coating exhibited a continuous diamond layer with diamond grains up to 80 nm in size.

The gas-sensing properties of the hydrogenated NCD sensor with a dense electrode arrangement of 50 µm was also tested against a sequence of NH_3_ pulses (Figure 2b). It is evident that after being exposed to NH_3_ at room temperature, the sensor response is slightly higher than that of the sensor with sparse electrode arrays (i.e., 200 µm; Figure 1b).

**Figure 3:**
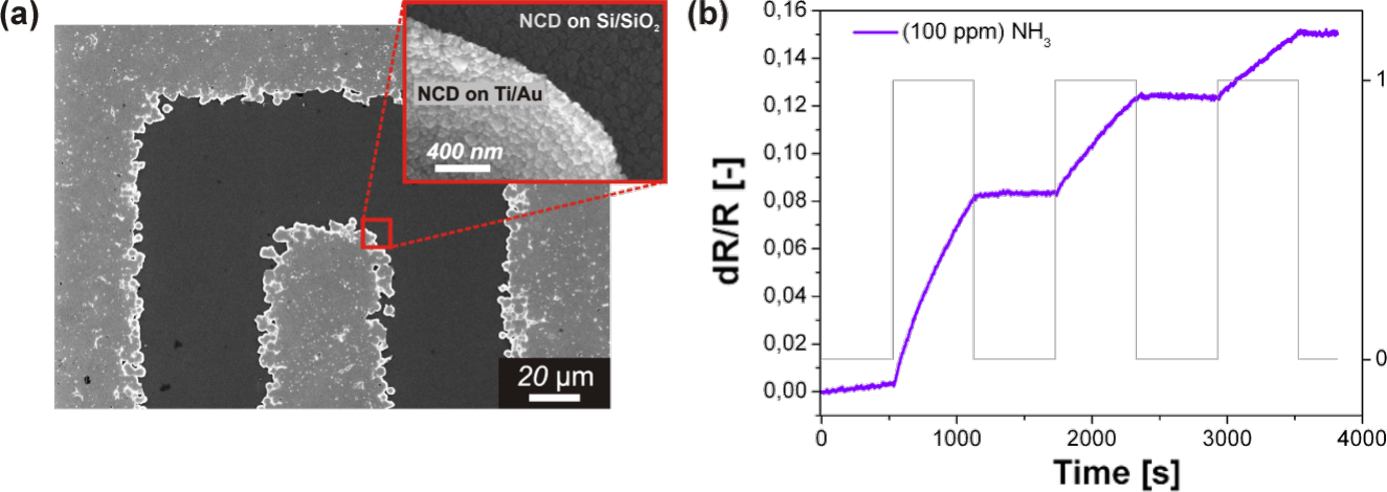
(a) SEM surface morphology of an NCD-coated sensor substrate with IDEs with separation of 50 µm and a nucleation time of 2 min, and (b) corresponding plot of the sensor response vs time.

Figure 3a shows an SEM image of a sample that was nucleated for 2 min. A homogenous and continuous NCD film is observed. Figure 3b shows the plot of the sensor response as a function of time for a pulsed sequence of 100 ppm NH_3_. The sensor displayed a significantly more rapid sensor response compared with the previous sensor (Figure 2b) for the same NH_3_ exposure. Again, clear evidence of the integrator property was observed.

Overall, the hydrogenated diamond sensors exhibited a response to each sequence of NH_3_. These behaviors indicated that the H-terminated NCD sensors were able to accumulate NH_3_ gas in its water adsorbate layer, which confirms the integrator-type gas sensor property. Moreover, the NCD sensor with the most dense electrode arrangement of 50 µm and a nucleation time of 2 min (Figure 3) exhibited the highest response toward NH_3_ gas. A similar behavior was observed by Beer et al., wherein the resistance at room temperature decreased or increased due to the electrolytic dissociation of the gases in the H-terminated diamond layer [3].

### Cold plasma microwave PECVD system

**Figure 4:**
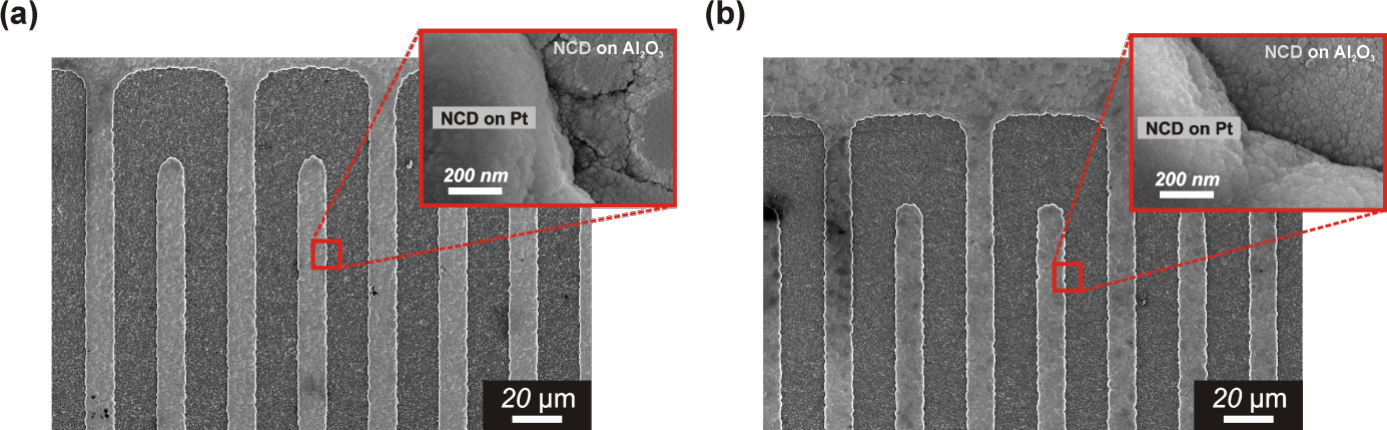
SEM images of NCD-coated, fully-integrated sensor substrates on a micro-hotplate with IDEs (separation of 15 µm) and a seeding time of (a) 1 min and (b) 5 min.

In the following experiments, the fully-integrated sensor device on a micro-hotplate was used. In contrast to hot plasma, where the plasma is localized close to the substrate surface (i.e., a distance of 1–2 mm), which may cause the substrate to overheat, a linear antenna, microwave plasma, CVD system (i.e., cold plasma) was used to avoid this drawback. The main advantage of a cold plasma system is the minimization of overheating of the substrate surface due to the longer distance between the substrate and the linear antenna (i.e., 7–10 cm) [11]. An NCD film was grown from hydrogen-rich gas mixtures of methane and carbon dioxide. A microwave pulse of 2 kW was used for each antenna side. A total gas pressure of 0.1 mbar was used and the substrate temperature was kept at 450 °C. Figure 4 shows the surface morphologies of fully-integrated sensor substrates coated with hydrogenated NCD films. Unfortunately, the SEM images also show that for the different nucleation times of 1 and 5 min, almost no differences were observed between the diamond morphologies. Both sensor substrates demonstrated a relatively smooth and continuous diamond film consisting of ultra-small grains (i.e., <50 nm).

**Figure 5:**
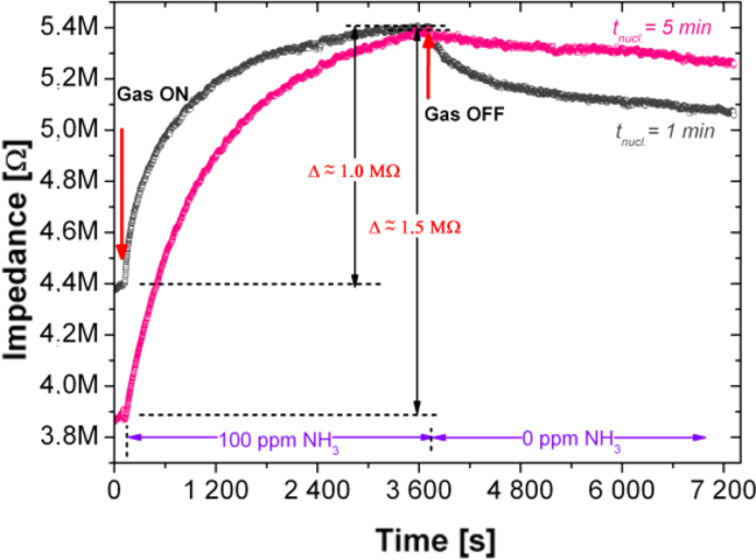
Time dependence of the impedance of fully-integrated sensor substrates coated with NCD; IDEs separation was 15 µm and the nucleation time (*t*_nucl_) was 1 or 5 min.

Figure 5 presents the room temperature response of fully-integrated sensor substrates (with 15 µm separation between IDEs) covered with H-terminated NCD with 1 and 5 min diamond nucleation times.

As illustrated in Figure 5, exposure of the sensor elements to 100 ppm of ammonia gas led to increased impedances from 4.4 to 5.4 MΩ and from 3.9 to 5.4 MΩ for the samples nucleated for 1 min and 5 min, respectively. It should be noted that a variation (shift) in the starting impedance value was observed. The starting impedance varied by nearly an order of magnitude in some cases. The origin for this difference can be attributed to several factors, for example, low quality ohmic contacts or memory effects of the surface state of NCD. It was concluded that technological optimization is still required for achieving better reproducibility and device reliability.

**Figure 6:**
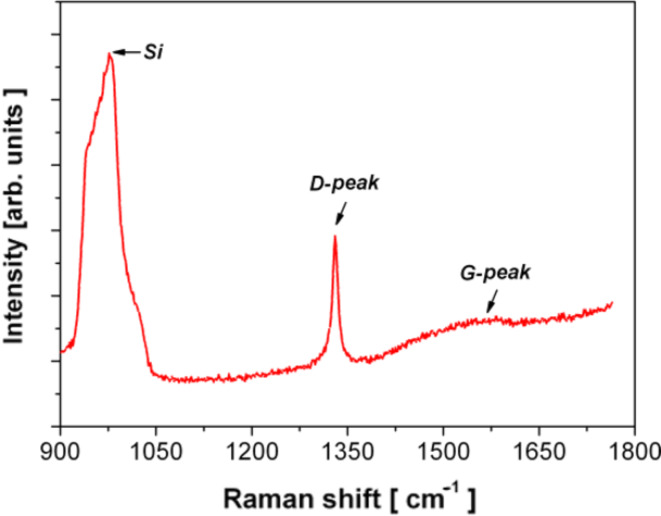
Raman spectrum of the NCD film. The sharp peak at 1330 cm^−1^ provides evidence of the diamond character of the deposited films.

The Raman spectrum of the structures (Figure 6) is characterized by two strong contributions: the peak characteristic for diamond centered at 1330 cm^−1^ (D-peak) and the broad band at approximately 1590 cm^−1^ attributed to the non-diamond phase (G-peak or sp^2^-bonded carbon atoms) [12].

**Figure 7:**
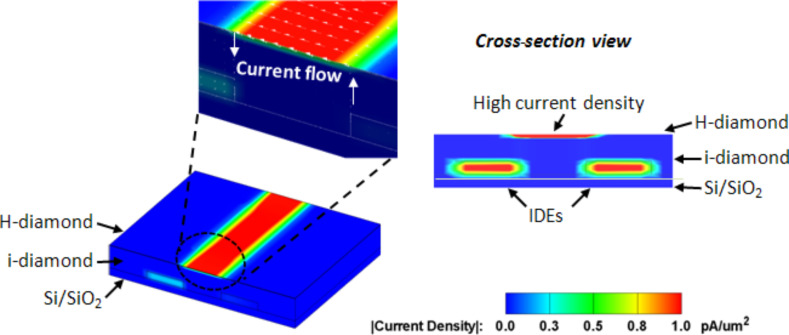
Current density profile for a single pair of IDEs covered by intrinsic diamond with a hydrogenated surface.

To determine the distribution of the electric field and the current density in the vicinity of the IDEs, a three-dimensional (3D) model was simulated. The results are shown in Figure 7.

The simulation of the current flow of the hydrogenated NCD was performed using the electrical solver MemPZR in the software package (Coventor, Raleigh, NC, USA). The insulating SiO_2_ substrate with Au IDEs (of 100 nm thickness) was covered by intrinsic diamond (i-diamond) at a thickness of 300 nm. The hydrogenated surface was modeled as an additional layer on top of the i-diamond at a thickness of 5 nm [8]. The model was discretized by a hexahedron parabolic mesh with over 19,000 elements. The interdigitated electrode arrangement was simplified to a single pair and the voltage boundary conditions were applied to the opposite sides of the metal electrodes/stripes (high voltage = 1 V, low voltage = 0 V). As demonstrated by the current density vectors in Figure 7, the simulation showed that current flowed from a high potential through the i-diamond to the H-diamond layer and then returned to the low potential. The maximum current density occurred on the surface area localized between the two conductive electrodes (Figure 7, cross-sectional view).

Moreover, the impedance measurements showed that the gap between the interdigitated electrodes is one of the most important geometric parameters of the sensor and should carefully be considered when enhancing the sensing response (Figure 3 and Figure 5) [13]. Furthermore, clear evidence of the integrator property of H-terminated NCD sensors was also demonstrated by the staircase-like increase of the sensor resistance (sensor response).

**Figure 8:**
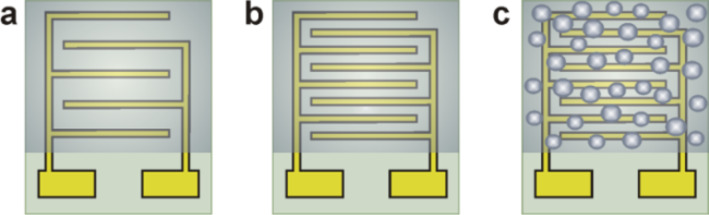
Schematic drawing of the sensor assemblies with diamond coatings: (a) continuous NCD film on IDEs with 200 µm electrode separation, (b) continuous NCD film on IDEs with 50 µm electrode separation, and (c) porous-like NCD film on IDEs with 50 µm electrode separation.

In our previous work, the morphology (porosity) of the diamond film was adjusted via seeding and/or growth time [9]. In the present work, a similar procedure was employed to achieve different diamond morphologies (Figure 8). However, the present results significantly differ from a recent study. The deposited nanocrystalline diamond layers formed under various nucleation times (i.e., 1, 2, 5 and 40 min) were found to be similar at a microscopic resolution. This could be due to the use of high concentration, water-based, diamond powder suspensions. Even when a short nucleation time of 1 min was used, a continuous diamond layer was formed (Figure 4a). This variation is a sign that the nucleation process is not well-controlled enough to reliably grow films of various porosities. However, the impedance measurements indicated a higher sensitivity for the sample nucleated using a short nucleation time of 2 min (Figure 3 b). Additionally, the AFM measurements have shown that at the nanoscale, the obtained diamond surface area was larger and was not well-resolvable by SEM measurements. The surface areas of the NCD films according to the samples presented in Figures 1, 2, and 3 were 1.20, 1.23, and 1.31 µm^2^, respectively*.*

The origin of the variation in the surface conductivity on the gas type has been the subject of a number of studies [14–16]. Overall, when oxidizing or reducing gases appear in the atmosphere, the charge exchange between the diamond and the adsorbed molecules causes an increase or a decrease in the conductance. The mechanism of this variation is broadly interpreted by the established surface transfer doping mechanism of the H-terminated diamond [17–18]. First, by exposing the H-terminated diamond surface to ambient air, a thin layer of adsorbed water is formed at the H-terminated surface. Based on the surface transfer doping model, Helwig et al. explained the sensitivity of the H-terminated NCD to NO_2_ and NH_3_ gases [1]. In their setup, the IDE electrodes were deposited on the top of the monocrystalline diamond. A high concentration of H_3_O^+^ ions was observed after exposure of the H-terminated diamond surface to NO_2_ gas because the electrons were transferred from the diamond sub-surface to the H_3_O^+^ species. Similarly, the increased surface conductivity of hydrogenated NCD upon exposure to phosgene gas was examined in one of our previous studies [2]. However, the exposure to NH_3_ gas led to a considerably lower density of H_3_O^+^ ions, which subsequently led to a decreased surface conductivity. Our results are in agreement with these observations. Additionally, in our case, the IDE electrodes were built into the diamond film, and the simulation confirmed that a closed current loop was formed between the IDEs and the H-terminated NCD surface.

## Conclusion

A nanocrystalline diamond film consisting of grains as small as 80 nm was used as the functional layer of a semiconductor gas sensor. Metallic electrodes were buried beneath the diamond film. This design protected them from harmful substances and the current flow localized at the grain boundaries. In this specific case, the H-terminated, diamond gas sensors behaved with an integrator-type gas response, that is, the sensor output signal was proportional to the integrated gas flow interacting with the diamond surface. The integrating properties were verified by cyclic measurements. The sensing characteristics of the H-terminated diamond layer towards NH_3_ gas were found to be dependent on the electrode arrangement (i.e., width gap). The sensor with a gap width of 50 µm exhibited a response twice as high as that of a sensor with an IDE separation of 200 µm. Moreover, the 3D model of the current density distribution of the hydrogenated NCD indicated the formation of a closed current loop, that is, the transient flow of electrons between the IDEs and the H-terminated diamond surface created a circular motion of the charge carriers. Additionally, the technological compatibility of the linear antenna plasma with a standard device confirmed that the functional NCD film can be deposited on its substrate without requiring other fabrication steps (e.g., lithography, masking, etc.). These results are promising for practical application in which small and simple H-NCD sensors can be used as gas sensors at room temperature.

## Experimental

A schematic view of the sensor assembly is shown in Figure 8. Two different sensor designs were used with a variety of metal interdigitated electrodes. The first sensor was fabricated in-house by standard UV lithography, thermal evaporation and lift-off techniques. For each pattern, six IDE electrodes were prepared with a separation of 50 or 200 μm. The second sensor was a commercially available product and consisted of a built-in micro-heater, a platinum temperature sensor, and a pair of interdigitated electrodes of 15 μm width.

Based on our previous study where the morphology (i.e., the porosity) of a diamond film was controlled via the seeding and/or growth time [9], a similar procedure was used in this present study to achieve different diamond morphologies (Figure 8). The sensor layer was based on the sandwich structure of the intrinsic H-terminated NCD layer/metal IDE/insulating substrate. The NCD growth proceeded in two steps: first, seeding for 1, 2, 5 or 40 min, followed by treatment by with microwave plasma-enhanced chemical vapor deposition (PECVD). The diamond layers were grown either by focused microwave PECVD (Aixtron P6, named as “hot plasma”) or pulsed-linear antenna microwave PECVD (Roth&Rau AK 400, named as “cold plasma”) [11,19–20]. Next, the samples were exposed to a pure hydrogen plasma for 5 min in order to generate a p-type surface conductivity [8,16] and then cooled down to room temperature. The resulting morphology of each structured NCD film was characterized by scanning electron microscopy (SEM, Raith *e*_LiNE). The diamond character of the sensor element was confirmed by Raman spectroscopy (Renishaw, In Via Reflex Raman spectrometer, 442 nm excitation wavelength).

For both sensor designs, the impedance measurements were realized at a voltage of 1 V and a frequency of 3 kHz (LCR—HIOKI 3532-50). A custom LabView program was used which allowed the temperature and gas-flow rate to be automatically controlled by a computer. Prior to the conductivity measurements, the sample was mounted in a gas-flow apparatus, and the chamber was flushed with dry nitrogen gas (N_2_) for 15 min to stabilize the output characteristics. Subsequently, the specific testing gas (NH_3_) was injected into the chamber through the inlet port, and the change in the resistance of the sensors (*dR*/*R*) was investigated as a function of exposure time. Ammonia (NH_3_, quality N38, purity 99.98%) was diluted with nitrogen (N_2_, quality N50, purity 99.999%) by mixing to the desired concentration. The sensor response was defined as a relative change in the resistance *dR = R − R*_o_ upon exposure to a specific gas (NH_3_) with respect to the resistance *R*_o_ (i.e., a change in the resistance *dR*/*R*) in the reference gas (N_2_).

## Supporting Information

File 1 Additional AFM and XRD experimental results.
