# Supplementary material for: Gas sensing properties of nanocrystalline diamond at room temperature
Source: Beilstein J Nanotechnol. 2014 Dec 4;5:2339–45. doi: 10.3762/bjnano.5.243 (PMC4273209; doi:10.3762/bjnano.5.243)
Supplement: File 1 — Additional AFM and XRD experimental results. [file Beilstein_J_Nanotechnol-05-2339-s001.pdf]

Supporting Information

for

Gas sensing properties of nanocrystalline diamond at room temperature

Marina Davydova[1*], Pavel Kulha[2], Alexandr Laposa[2], Karel Hruska[1], Pavel Demo[1] and Alexander Kromka[1]

Address: [1]Institute of Physics, Academy of Science of the Czech Republic, Cukrovarnicka 10, 16200 Prague, Czech Republic and [2]Department of Microelectronics, Faculty of Electrical Engineering, CTU in Prague, Technicka 2, 16627 Prague, Czech Republic

Email: Marina Davydova* - davydova@fzu.cz

* Corresponding author

Additional AFM and XRD experimental results

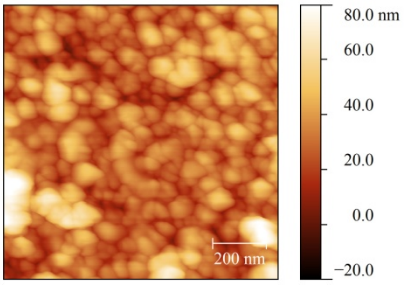

RMS = 10.7 nm
Surface area = 1.20 µm^2
(a)

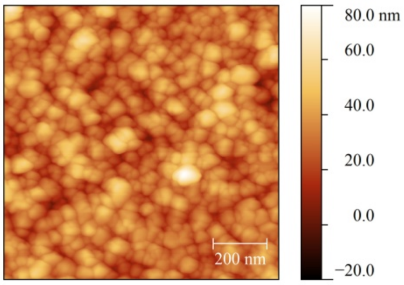

RMS = 8.7 nm
Surface area = 1.23 µm^2
(b)

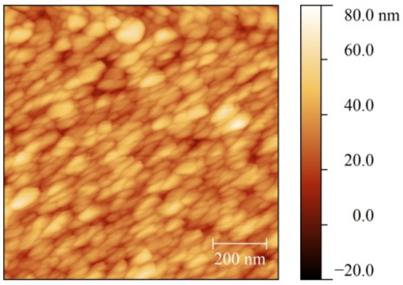

RMS = 8.9 nm
Surface area = 1.31 µm^2
(c)

Figure S1: AFM surface topography of the deposited NCD-coated sensor substrate: **(a)** with separation of 200 µm and nucleation time of 40 min **(b)** with separation of 50 µm and seeding time of 40 min, and **(c)** with separation of 50 µm and nucleation time of 2 min.

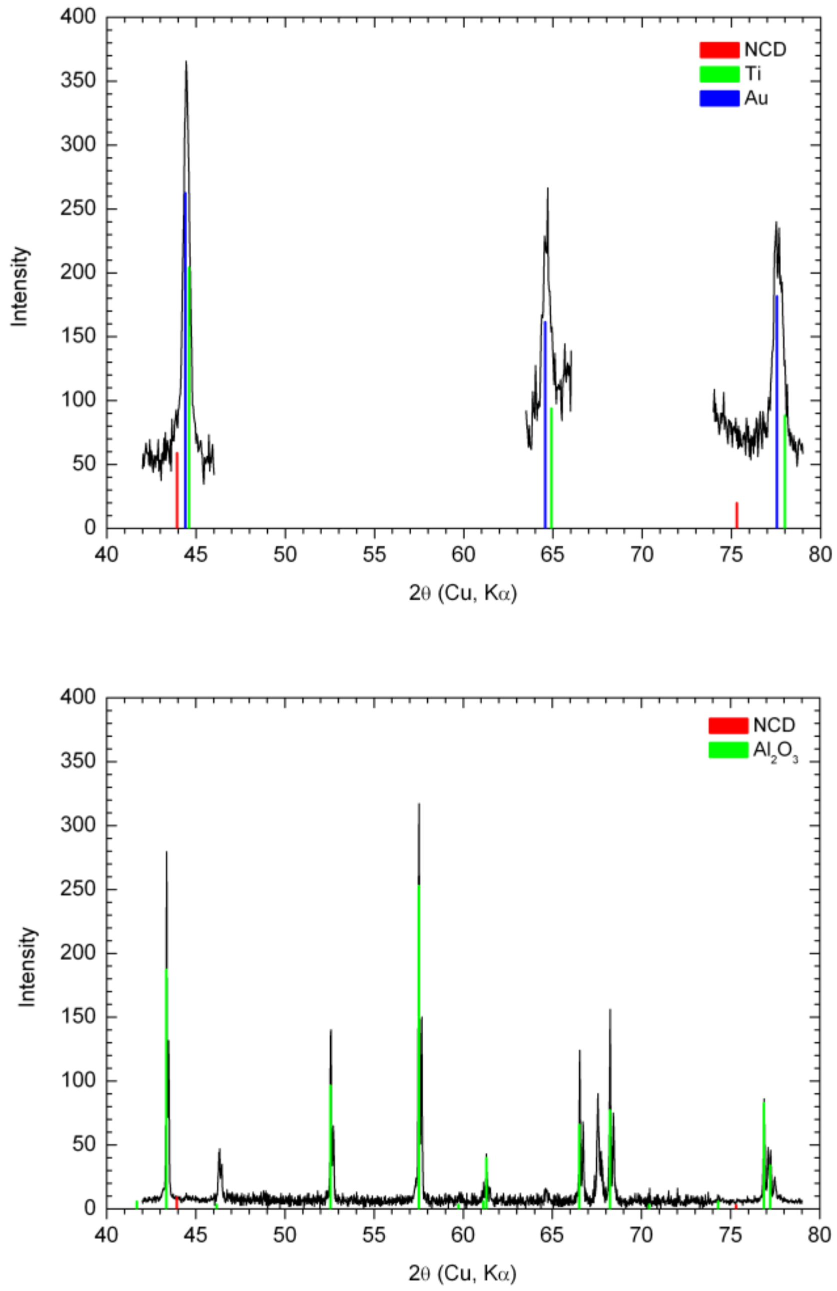

Figure S2: XRD patterns of sensor substrates covered with H-terminated diamond.
